# Special Issue “Molecular Research on Adenosine Receptors: From Cell Biology to Human Diseases”

**DOI:** 10.3390/ijms262311298

**Published:** 2025-11-22

**Authors:** Federica Mannino

**Affiliations:** Department of Medicine and Surgery, University of Enna “Kore”, Contrada Santa Panasia, 94100 Enna, Italy; federica.mannino@unikore.it

## 1. Adenosine Receptors: Central Molecular Regulators in Physiology and Human Disease

Adenosine receptors (ARs), members of the G protein-coupled receptor (GPCR) superfamily, have emerged as central regulators of numerous physiological and pathological processes. Their four subtypes—A1, A2A, A2B, and A3—play distinct yet interconnected roles in neurotransmission, cardiovascular function, immune modulation, and metabolic homeostasis [1,2,3]. Over the past three decades, adenosine receptor research has shifted from fundamental receptor pharmacology to translational studies exploring their therapeutic potential in cancer, inflammation, cardiovascular disease, and metabolic disorders [4,5,6].

This Special Issue, Molecular Research on Adenosine Receptors: From Cell Biology to Human Diseases, brings together recent advances that highlight canonical receptor functions and emerging molecular pathways linking ARs to inflammation, hemostasis, arrhythmogenesis, immunometabolism, and epigenetic regulation. The articles included showcase the versatility of AR signaling and the promise of AR-targeted therapies across a spectrum of human diseases.

## 2. Novel Compounds and Anti-Inflammatory Potential

The identification of novel compounds that modulate adenosine receptor activity is a rapidly expanding field, with significant implications for the treatment of inflammation-related disorders. A central contribution of this Special Issue comes from Azumi et al. [7], who investigated the organogermanium compound 3-(trihydroxygermyl)propanoic acid (THGP). Their work demonstrated that THGP exerts anti-inflammatory effects through the adenosine–NR4A2 signaling axis, highlighting a novel molecular pathway that could be harnessed for therapeutic development. This finding adds to the growing evidence that adenosine receptors, beyond their canonical roles in neurotransmission and cardiovascular regulation, serve as pivotal modulators of immune responses [8,9].

Complementing this study, Załuski et al. [10] characterized a series of 8-benzylaminoxanthines, which exhibited potent anti-inflammatory activities mediated by high affinity for adenosine A2A receptors and dual A1/A2A receptor binding. The dual activity is particularly intriguing, since simultaneous modulation of different receptor subtypes may enhance efficacy or reduce adverse effects in inflammatory diseases [3,11]. The chemical versatility of the xanthine scaffold, historically found in methylxanthines such as caffeine and theophylline, underscores its enduring value as a platform for drug discovery [12].

These advances converge with a broader body of research indicating that pharmacological manipulation of adenosine A2A receptors can attenuate excessive immune activation and tissue injury in models of arthritis, inflammatory bowel disease, and neuroinflammation [13,14,15]. Importantly, novel antagonists and agonists with refined receptor selectivity are being developed to optimize therapeutic outcomes and to minimize off-target effects such as hypotension or tachycardia [4]. Furthermore, emerging data suggest that biased agonism of adenosine receptors—whereby ligands preferentially activate specific intracellular pathways—may represent a transformative approach to harnessing anti-inflammatory benefits while avoiding unwanted signaling cascades [16,17].

Together, these studies provide compelling evidence that innovative small molecules, ranging from organogermanium derivatives to optimized xanthine-based compounds, can significantly expand the pharmacological repertoire targeting adenosine receptors. Such compounds may pave the way for next-generation anti-inflammatory therapies, particularly in conditions where current treatments fail to achieve durable remission or carry substantial side effects.

## 3. Antiplatelet Effects and Cardiovascular Implications

Adenosine receptors, particularly the A2A and A2B subtypes, play a crucial role in regulating platelet function and cardiovascular protection [3,4,18]. Kubacka et al. [19] demonstrated that certain selective xanthine-based antagonists modulate platelet activity in rat experimental models, suggesting that pharmacological modulation of A2A and A2B receptors can influence thromboinflammatory processes. These findings are clinically relevant, as excessive thrombosis and platelet aggregation are key factors in acute cardiovascular events such as myocardial infarction and ischemic stroke [20,21].

A2A receptor activity is particularly important for the regulation of coronary vasodilation and inhibition of platelet aggregation [22]. Preclinical studies have shown that A2A activation reduces endothelial leukocyte adhesion and local vascular inflammation, contributing to myocardial protection during ischemia-reperfusion injury [14]. Similarly, A2B receptor activation can modulate inflammatory responses and vascular remodeling, although its role may be context-dependent depending on the tissue microenvironment [23].

From a translational perspective, selective modulation of A2A and A2B receptors represents a promising strategy for developing antithrombotic and cardioprotective therapies with a more favorable safety profile compared to traditional anticoagulants, which carry a significant bleeding risk [24,25]. Moreover, the integration of biased ligands offers the possibility of selectively activating protective pathways while minimizing systemic side effects [16].

In summary, these studies highlight how adenosine receptor pharmacology may open new avenues for thrombosis management and cardiovascular protection, emphasizing the importance of understanding receptor subtype selectivity and the pathophysiological context.

## 4. Receptor Density in Pathophysiology: Atrial Fibrillation as a Case Study

Alterations in adenosine receptor expression and density are increasingly recognized as key contributors to cardiovascular pathophysiology. A2A receptors, in particular, have been implicated in atrial electrophysiological remodeling, arrhythmogenesis, and myocardial inflammation [3,18,20]. Godoy-Marín et al. [26] reported a significant increase in endogenous A2A receptor density in atrial tissue from patients with atrial fibrillation (AF), as well as in cellular and porcine models. This upregulation was associated with enhanced cAMP signaling and increased susceptibility to arrhythmic triggers, suggesting that receptor density directly modulates electrical stability in the atrium.

These findings align with previous studies showing that A2A receptor overexpression can accelerate spontaneous calcium release and afterdepolarizations, both of which are mechanistic hallmarks of AF [27,28]. Furthermore, the differential distribution of A2A and A2B receptors within atrial myocytes and fibroblasts implies a complex interplay between electrophysiological and fibrotic remodeling processes. For instance, activation of A2A receptors in fibroblasts promotes pro-fibrotic signaling via cAMP/PKA pathways, which may contribute to structural remodeling and maintenance of AF [29].

From a translational perspective, these results highlight the potential of targeting receptor density and function as a therapeutic strategy. Modulation of A2A receptor signaling could stabilize atrial electrophysiology while attenuating inflammatory and fibrotic processes, offering an alternative or complementary approach to conventional antiarrhythmic drugs and catheter ablation [30,31]. In addition, understanding receptor distribution at the cellular and tissue level may help predict patient responsiveness to adenosine-based pharmacotherapies and guide precision medicine approaches in AF management.

Finally, this case study underscores a broader principle: changes in receptor density, not just receptor activation, are critical determinants of pathophysiology. Similar phenomena are observed in other conditions, such as heart failure, ischemia-reperfusion injury, and vascular inflammation, where AR upregulation or downregulation modifies cellular signaling and disease progression [32,33,34].

## 5. Non-Canonical Functions and Expanding Horizons

Beyond their classical roles in modulating cAMP and calcium signaling, adenosine receptors (ARs) are increasingly recognized for their non-canonical functions, encompassing metabolism, immunometabolism, and epigenetic regulation [3,4,15]. Pallio and Mannino [35] highlighted how ARs integrate signaling pathways that go beyond the classical G-protein-mediated mechanisms, affecting transcriptional networks, chromatin remodeling, and cellular metabolism. These emerging roles provide a conceptual framework to understand the pleiotropic effects of ARs in health and disease.

For example, A2B receptors have been shown to regulate adipose tissue expansion and glucose homeostasis. In murine models, A2B activation modulates adipocyte differentiation and insulin sensitivity, linking purinergic signaling to metabolic health [36,37]. Similarly, A2A receptor activation in macrophages promotes an anti-inflammatory M2 phenotype, demonstrating a direct connection between AR signaling and immunometabolic adaptation [6,9]. These effects underscore the potential of targeting ARs in metabolic diseases such as obesity, type 2 diabetes, and non-alcoholic fatty liver disease.

Recent studies have also unveiled epigenetic dimensions of AR signaling. Activation of ARs can alter histone acetylation, DNA methylation, and microRNA expression, thereby regulating gene expression in a cell-type-specific manner [38,39]. This suggests that AR ligands may not only produce acute functional effects but also induce longer-term modifications in cellular behavior, with implications for chronic inflammation, cancer, and cardiovascular disease. For instance, the A2A receptor has been implicated in the epigenetic modulation of cytokine gene expression in T cells and dendritic cells, offering a mechanistic link between receptor activation and immune tolerance [8].

Moreover, non-canonical AR functions extend to the regulation of autophagy, oxidative stress, and mitochondrial dynamics, connecting purinergic signaling to fundamental aspects of cellular homeostasis [32,33]. By influencing these intracellular processes, ARs act as integrators of metabolic and stress signals, coordinating adaptive responses to inflammation, ischemia, and nutrient stress.

In summary, the expanding landscape of AR biology reveals versatile roles that go far beyond classical GPCR signaling, integrating metabolic, immunological, and epigenetic regulatory networks. These insights provide a rich foundation for the development of innovative therapeutic strategies that leverage both canonical and non-canonical pathways.

## 6. Translational Relevance and Future Perspectives

The cumulative evidence presented in this Special Issue highlights the translational potential of adenosine receptor research, spanning from molecular insights to human diseases. Novel compounds targeting A1, A2A, and A2B receptors demonstrate promising anti-inflammatory, antiplatelet, and cardioprotective effects, suggesting new therapeutic avenues for conditions ranging from atrial fibrillation and thrombosis to metabolic and inflammatory disorders [7,10,19,26]. By integrating classical and non-canonical functions of ARs—including immunometabolic regulation, epigenetic modulation, and cellular homeostasis—these studies pave the way for more precise, mechanism-based interventions.

A critical future direction is the development of selective and biased ligands, which can preferentially activate beneficial signaling pathways while minimizing adverse effects. Biased agonism represents a particularly exciting strategy, allowing the dissociation of anti-inflammatory or cardioprotective effects from unwanted hemodynamic or arrhythmogenic consequences [16,17]. Additionally, emerging data on receptor density alterations and tissue-specific expression underscore the importance of personalized approaches: patient stratification based on receptor profiling could optimize therapeutic outcomes in cardiovascular, metabolic, and immune-mediated diseases [18,33].

Another promising area is the integration of AR-targeted therapies with multi-modal strategies, including lifestyle interventions, metabolic modulators, and complementary pharmacological agents. For example, combining selective AR agonists with anti-inflammatory diets or exercise regimens could enhance efficacy in metabolic disorders, while minimizing drug doses and side effects [6,9]. Similarly, AR modulation could synergize with existing cardiovascular therapies, such as anticoagulants or antiarrhythmics, offering additive or even synergistic protection [20].

Finally, the elucidation of non-canonical and epigenetic AR functions opens new frontiers for drug discovery and disease prevention. Modulating AR-mediated transcriptional programs, autophagy, and mitochondrial dynamics could provide innovative therapeutic strategies in chronic diseases, cancer, and immune dysfunction [32,35,38]. As molecular tools, animal models, and human studies continue to advance, adenosine receptors stand out as versatile and clinically relevant targets, bridging fundamental cell biology and translational medicine.

In conclusion, the insights gained from the studies in this Special Issue not only deepen our understanding of AR biology but also highlight a roadmap for future translational research, emphasizing the importance of integrative approaches that consider receptor subtype specificity, tissue context, and non-canonical signaling. Such strategies hold the promise of developing more effective, safer, and personalized therapies for a broad spectrum of human diseases.
